# Nanoscale solid-fluid interaction and amphibole formation in the lithospheric mantle

**DOI:** 10.1038/s41598-026-40179-1

**Published:** 2026-02-17

**Authors:** Thomas Pieter Lange, Mihály Pósfai, Márta Berkesi, Péter Pekker, Zsófia Pálos, Gábor Molnár, Csaba Szabó, István János Kovács

**Affiliations:** 1 HUN-REN Institute of Earth Physics and Space Science, Sopron, Hungary; 2 MTA - HUN-REN FI FluidsByDepth Momentum Research Group, Sopron, Hungary; 3 Lithosphere Fluid Research Lab, Institute of Geography and Earth Sciences, Eötvös Loránd University, Budapest, Hungary; 4 HUN-REN–PE Environmental Mineralogy Research Group, Veszprém, Hungary; 5 Research Institute of Biomolecular and Chemical Engineering, University of Pannonia, Veszprém, Hungary; 6 Mineral Resources and Geofluids Group, Department of Earth Sciences, University of Geneva, Geneva, Switzerland; 7 Alba Regia Faculty, Institute of Geoinformatics, Óbuda University, Székesfehérvár, Hungary; 8 MTA FI Pannon LitH2Oscope Momentum Group, Sopron, Hungary

**Keywords:** Amphibole, Nanochannel, Fluid inclusion, Lithospheric mantle, Perșani mountain mantle xenoliths, Monolayer, Geochemistry, Mineralogy

## Abstract

Nanoscale amphibole nucleation and growth in the Earth’s mantle is poorly understood despite its important role in the evolution and stability of the lithosphere, along with its influence on the coexisting fluid composition. Here, we focus on the nanoscale features of a clinopyroxene-amphibole-fluid inclusion system in an amphibole-bearing upper mantle xenolith and propose a new amphibole formation mechanism at mantle depth. Our approach includes crystallographic observations and interpretations of fluid inclusion formation and molecular properties of the entrapped H_2_O-bearing supercritical CO_2_-rich mantle fluids. First, the differentiation of H_2_O and CO_2_ along the supercritical fluid-clinopyroxene interface leads to nanometre-thick hydrous monolayer formation. The disruption of the supercritical fluid-clinopyroxene interface, including the hydrous monolayer, leads to amphibole formation. Hydrous fluid-mobile elements in the hydrous monolayers support amphibole growth via diffusion along the clinopyroxene-amphibole interface enhanced by various structural misfits. Our results help to clarify the driving forces of element exchange in a supercritical fluid-solid system and present the initial stage of hydrous mineral formation and growth. This process causes fixation of H_2_O and common lithophile elements with relative CO_2_ enrichment in the residual supercritical fluid. Nanochannels can contribute to element migration in clinopyroxene-amphibole-rich, non-porous domains in the lithospheric mantle.

**Supplementary Information:**

The online version contains supplementary material available at 10.1038/s41598-026-40179-1.

## Introduction

Fluid-rock interactions have a profound effect on geological processes at different horizons within the Earth. Small-scale observations of terrestrial materials, including both solid and fluid state materials, provide insight into molecular reactions, potentially affecting large-scale processes (e.g., subduction, mantle metasomatism, deep fluid degassing) and facilitate to recognise phenomena such as solid-fluid interaction at the nanoscale^1–3^. Thus, fluid infiltration, phase transformation, dissolution and reprecipitation, surface energy minimalization, dielectric permittivity and elemental/molecular diffusion can be better understood^2,4,5^. Nanoscale fluid infiltration along interfaces or grain boundaries is mostly influenced by fluid pressure, chemical gradient and surface potential^1^. This is followed by the formation of few-nanometre-thick monolayers along the solid-fluid interface due to different molecular polarities and differing nanopore size (e.g.^3,4,6,7^). As a result, fluid complexes, which are often carried by the solvent^8^, can be adsorbed or absorbed forming solid-like structure with compositions distinct from that of the bulk fluid^9^. Thus, monolayers, structurally, act as intermediaries between the solid and fluid phases that can enhance mineral dissolution-reprecipitation and mineral growth^4,10^.

In the lithospheric mantle, modal metasomatism is the key driving process for element transport by fluid infiltration, and triggers phase transformation via dissolution-reprecipitation reactions resulting in rheological and chemical horizons within the lithosphere^11,12^. Nanoscale observations of phase transformation, nucleation and growth during modal metasomatism are available mostly for lower crustal lithologies, mainly related to metamorphic processes^2^. In the lithospheric mantle, nanoscale studies were dominantly applied to reveal mantle rheology^13,14^, whereas nanoscale studies of solid-fluid interactions for understanding hydrous mineral formation are scarce^3,5^. At elevated temperature and pressure conditions (e.g., in the lower crust and upper mantle), volatile-rich fluids are supercritical and act differently from near-surface (low P-T) fluids^15,16^. Volatile-rich supercritical fluids in the lithospheric mantle mainly consist of C, O and H (+ N and S)^17^, and can get trapped and be directly studied as high-density, negative crystal-shaped fluid inclusions in rock-forming silicate minerals^15,17^. These supercritical fluids can contain dissolved NaCl, which enhances the solubility of silicate components (like Si, Al^18^). Negative crystal shape forms through the dissolution and reprecipitation of the host mineral, in the presence of the entrapped fluid and, likely, a distinct monolayer at the surface^1^. The process is governed by the surface energy minimization^19^. Amphibole formation by the interaction of supercritical fluid with clinopyroxene (single- to double-chain silicate transformation) was intensely studied experimentally^20–25^, yet only few studies discussed the geochemical consequences of nanoscale structural changes^26^. One of such consequences is the incorporation of hydrogen from the fluid into the solid, which leads to the relative enrichment of incompatible components in the solid phases and the formation of more evolved fluids, such as CO_2_-rich mantle supercritical fluids above ~ 70 km depth^27–29^) that significantly differ in their fluid molecular properties (e.g., distribution and interactions of dissolved species) compared to the initial supercritical fluids.

**Fig. 1:**
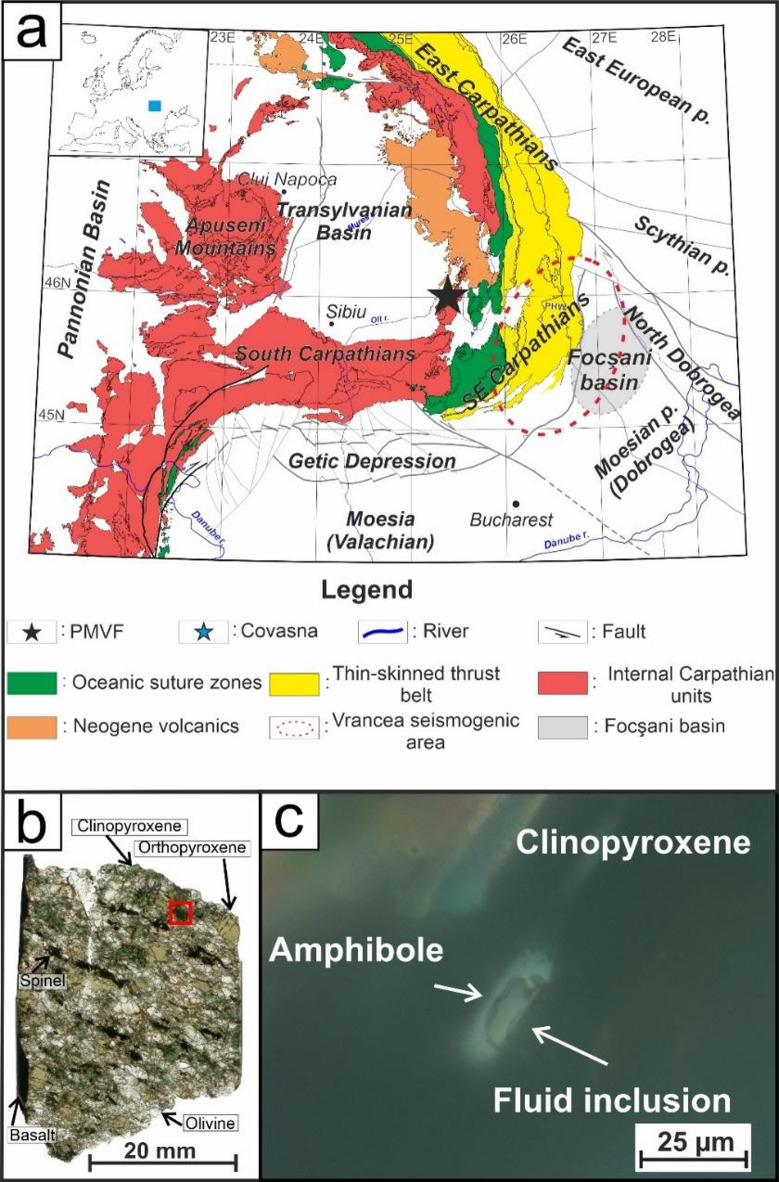
(**a**) Schematic geological map showing the main units of the Transylvanian Basin, the East, Southeast and South Carpathians. Black star shows the location of the Perșani Mountains Volcanic Field, where the studied slightly deformed lherzolite xenolith originates from. (**b**) Thick section of the PGR-X1-0345 lherzolite xenolith which hosts the fluid inclusion and associated amphibole of this study. (**c**) Optical photomicrograph of the clinopyroxene that contains the amphibole of interest (the area shown is marked by the red square in (**b**)). IMF = Intra-Moesian Fault; TF = Trotus Fault; NTF = New Trotus Fault; PCF = Peceneaga-Camena Fault. (**a**) Is modified after^30^ with the use of CorelDRAW Graphics Suite 2024 (https://www.coreldraw.com/en/). The thin section of (**b**) is scanned with an HP Scanjet 2400 scanner with a resolution of 3200 dpi and modified with the use of CorelDRAW Graphics Suite 2024 (https://www.coreldraw.com/en/). The photomicrograph of **c** is made with a Nikon DS-Fi1 digital camera attached on a Nikon Eclipse LV100 POL polarizing microscope. The image was processed with NIS Elements AR 2.20 digital imaging software and later modified with the CorelDRAW Graphics Suite 2024 (https://www.coreldraw.com/en/).

Here, we use a joint geochemical and mineralogical approach to better understand the nanoscale processes of the clinopyroxene to amphibole phase transformation at lithospheric mantle depth. We present results for a system consisting of volatile-rich fluid inclusions, host clinopyroxene and fluid inclusion-associated amphibole^30^, within a slightly deformed amphibole-bearing lherzolite xenolith from the locality Gruiu of the Neogene Perșani Mountains Volcanic Field (Carpathian-Pannonian region, Central Europe) (Fig. 1, more details in Supplementary Material;^30^). Amphibole occurs in several types: as interstitial grains, as exsolution lamellae, and as fluid inclusion-associated crystals, all with similar major element composition (^30^; Supplementary Material). The present study is concerned mainly with the fluid inclusion-associated type amphibole. Our findings also help to better understand interstitial amphibole formation and suggest a clear relationship between the interface structures of volatile-rich, supercritical fluid and clinopyroxene, and that of clinopyroxene and amphibole. At these interfaces, hydrous fluid complexes can have a more significant role than previously expected. Nanoscale chemical zoning in the supercritical fluid along the fluid-clinopyroxene interface triggers fluid-solid interaction and highlights the significance of hydrous monolayers and the maturation of the fluid-solid interface, a process that is governed by mineral dissolution and precipitation^31,32^. Our model presented here contributes to the understanding of fluid-mediated reactions in the lithospheric upper mantle, taking place simultaneously with the maturation of the shapes of entrapped fluid inclusions. The mechanisms, shown in this study, provide a new nanoscale aspect on global volatile element cycling, specifically how mantle metasomatism and fluid migration takes place in the lithospheric mantle. These processes clearly explain the non-volcanic origin of mantle degassing^33,34^.

## Methods

We prepared 100 nm thick lamellae for transmission electron microscopy (TEM) across clinopyroxene-amphibole interfaces, perpendicular to the [001] axes of both structures, using focused ion-beam scanning electron microscopy (FIB–SEM) at the Research and Instrument Core Facility of Eötvös Loránd University, Faculty of Science (Hungary) (Supplementary Material). The TEM analysis was performed using a Talos F200X G2 scanning transmission electron microscope (Thermo Fisher) at the Nanolab of the University of Pannonia (Hungary) (Supplementary Material).

**Fig. 2:**
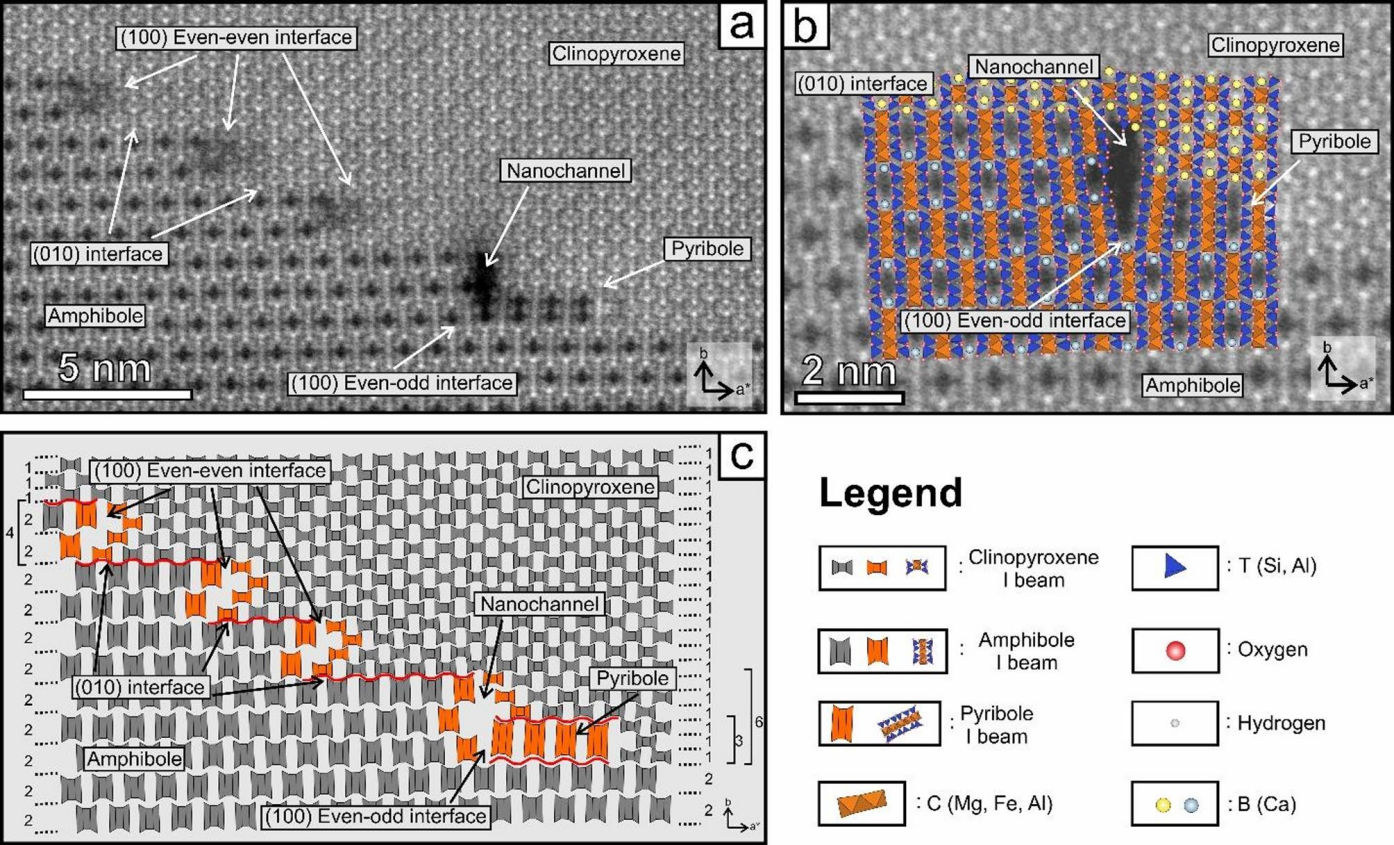
(**a**) High-angle annular dark-field scanning transmission electron microscopy image of clinopyroxene-amphibole interfaces. (**b**) Structural interpretation of the nanochannel marked in (**a**). (**c**) Structural model of the area in (**a**) using the I-beam representation.

To characterize the atomic structure of clinopyroxene and amphibole interfaces, we obtained high-angle annular dark-field (HAADF) images in scanning (STEM) mode, with [001] of both clinopyroxene and amphibole oriented parallel to the electron beam. Figure 2 shows individual atom column positions within the ‘I-beams’, i.e., the projected chains of [SiO_4_] tetrahedra and [MO_6_] octahedra between them^35^. Since in HAADF images the contrast is directly proportional to the square of the atomic number, the brightest spots in the images correspond to the positions filled by Ca^2+^ ions (M2 in pyroxene and M4 in amphibole), whereas the other cation positions appear as less intense spots.

Amphibole formed exclusively after fluid entrapment, and its formation was initiated by the reaction of the supercritical fluids, entrapped in the fluid inclusion, with the host clinopyroxene^30^. In this regard, we modelled the initial H_2_O-content of the entrapped and reacting supercritical fluid by combining the molar H_2_O content of various amphibole lamellae and adjacent fluid inclusion pairs by mass balance calculation (Supplementary Material). For simplification, we assumed (1) the amphibole to be pure OH-amphibole (i.e., Cl and F-free), (2) the clinopyroxene being free of structural OH, and that (3) the entrapped supercritical fluid represents a CO_2_-H_2_O system. Fluid inclusions contain CO_2_-rich supercritical fluids based on the high CO_2_/(other fluid species) molar ratio (> 80 mol%) and are present in wide pressure and temperature ranges in the lithospheric mantle^27,30^. In addition, the large difference in Na_2_O and Al_2_O_3_ contents between the host clinopyroxene and newly formed amphibole^30^ provides an opportunity to estimate minimum Na_2_O and Al_2_O_3_ contents of the initial, entrapped supercritical fluid. Details regarding the modelling are described in Supplementary Material. The data used for the description are presented in Supplementary Tables S1–4.

## Results

**Fig. 3:**
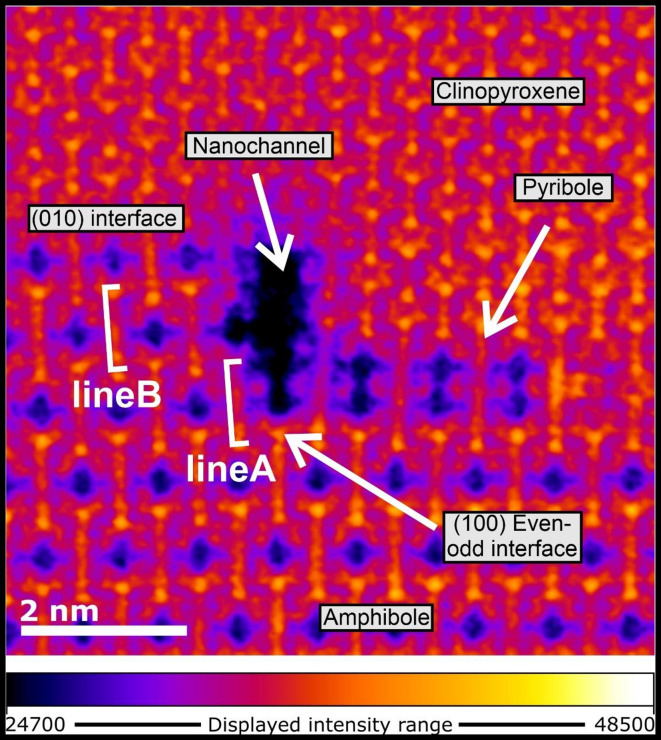
False-colour image of a nanochannel and its surroundings, calculated from the pixel intensities of an atomic-resolution high-angle annular dark-field image. The octahedral positions bordering the nanochannel (line A) display lower intensity compared to similar positions further away from the nanochannel (line B); a similar decrease in intensity is observed for the tetrahedral positions. Based on the assumed lack of significant compositional changes in tetrahedral positions, the decrease in pixel intensity towards the nanochannel can be attributed to a reduction in thickness; the walls of the nanochannel are not flat on the atomic scale through the entire sample thickness.

In general, no structural defects occur in the bulk clinopyroxene and amphibole, therefore, a robust fit along the (010) interface between the two minerals can be observed (Fig. 2; Supplementary Material). In contrast, the (100) clinopyroxene-amphibole interface shows abundant structural defects, displaying two types of structural matchings (Fig. 2). As described by Veblen and Buseck^26^, in places where either even/even or odd/odd numbers of I-beams occur on both sides of the (100) interface, the two structures show almost perfect fit (Fig. 2). In contrast, structural defects are more abundant at interfaces with even/odd numbers of I-beams on opposite sides of the interface (Fig. 2; Supplementary Material; Supplementary Fig. S1 and S2). We observed three types of even/odd interfaces. The most frequent even/odd interface is the 6 clinopyroxene to 3 amphibole (6c-3a) I-beam transformation that is associated with an empty volume (hereafter referred to as ‘nanochannel’) and is the focus of this study (Fig. 2). Here, the I-beams surrounding the ‘nanochannel’ are inclined relative to the bulk crystal, and their degree of inclination decreases with distance from the nanochannel (Fig. 2). Adjacent to the (100) clinopyroxene side of the ‘nanochannel’, a 3-clinopyroxene-wide I-beam, a pyribole (hereafter clinojimthompsonite^36^; can be observed with similar inclination to the adjacent clinopyroxene and amphibole I-beams. We observed decreasing intensity representing atomic positions in the HAADF images in the I-beams along the nanochannel boundary, probably related to geometrical variations along the length of the channel (Fig. 3; Figs. S3–5). We also observed 2 clinopyroxene to 1 amphibole, and 10 clinopyroxene to 5 amphibole even/odd clinopyroxene-amphibole interfaces that are described in detail in Supplementary Material. Furthermore, following the (010) clinopyroxene-amphibole interface, a slight bend towards the [010] direction of the (010) interface can be observed for both even/even and even/odd clinopyroxene-amphibole interfaces (Supplementary Material).

Mass balance calculations to determine the initial (pre-amphibole) H_2_O content of the entrapped supercritical fluid always show CO_2_-rich characteristics (**>** 85 mol%, Supplementary), regardless of the amount of the H_2_O reintegrated during the calculation from the amphibole into the residual supercritical fluid (Supplementary). Nevertheless, the mass balance calculation results assume that H_2_O mol% of the initially entrapped supercritical fluid was up to several 10% higher than the H_2_O mol% of the residual supercritical fluid (Supplementary). In other words, amphibole formation significantly decreases the initial H_2_O-content of the supercritical fluid, regardless of the initial H_2_O mol% within the fluid inclusion but it does not change the dominant component (i.e., CO_2_).

## Discussion

### Pre-amphibole state and amphibole formation

**Fig. 4:**
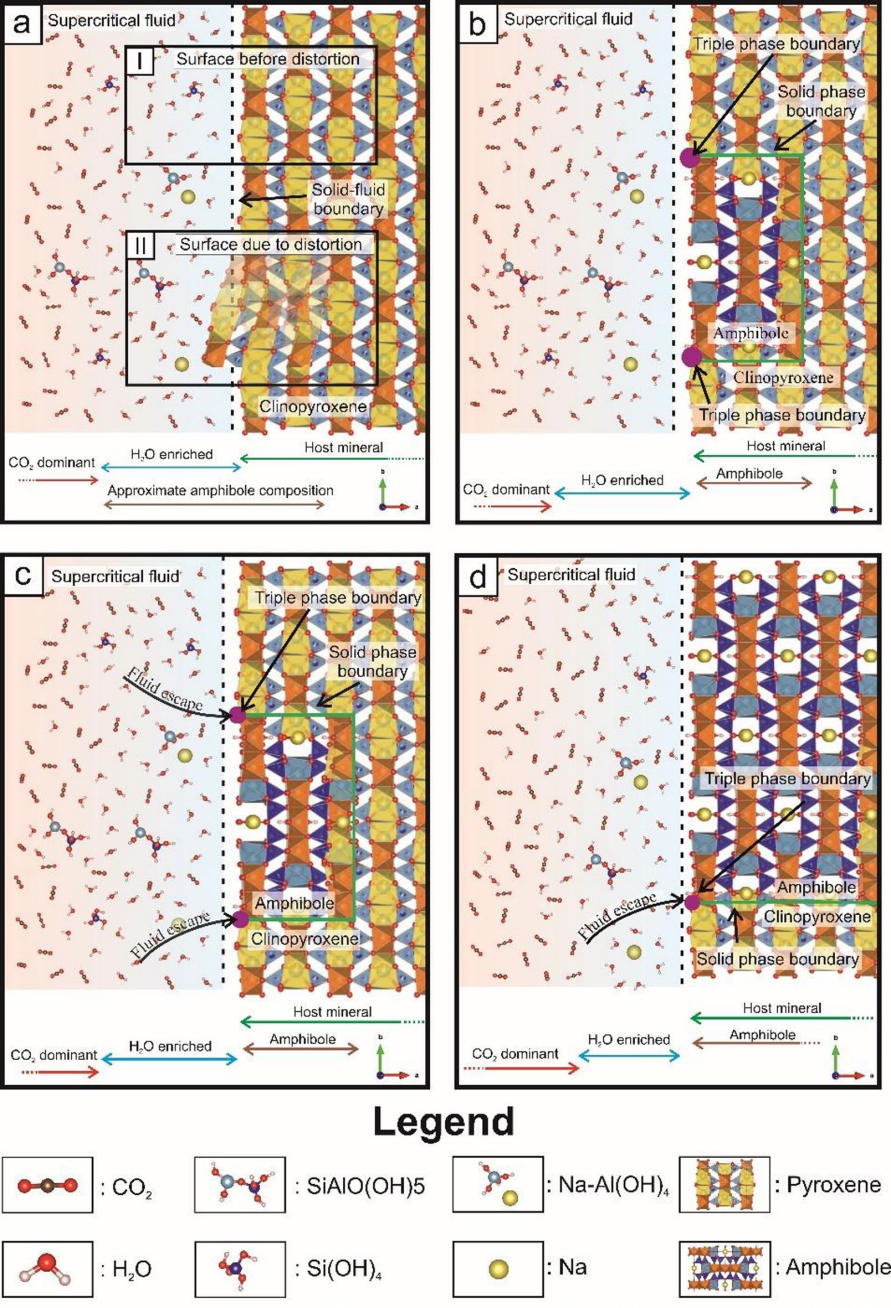
Nanoscale amphibole formation, viewed from [001], at the interface of a secondary, negative crystal-shaped fluid inclusion and its host clinopyroxene. (**a/I**) In the initial stage the interface between the fluid inclusion and clinopyroxene is intact, enclosing an H_2_O-enriched layer (i.e., hydrous monolayer). (**a/II**) In the next stage, surface distortion initiates dissolution of the clinopyroxene, (**b**) followed by amphibole reprecipitation. (**c**) The newly formed clinopyroxene-amphibole-fluid triple junction provides a pathway for fluid loss into the clinopyroxene-amphibole interface, (**d**) supporting amphibole growth with a simultaneous decrease in the width of the hydrous monolayer. Symbols for atom positions within the solid structures are the same as in Fig. 2.

**Fig. 5:**
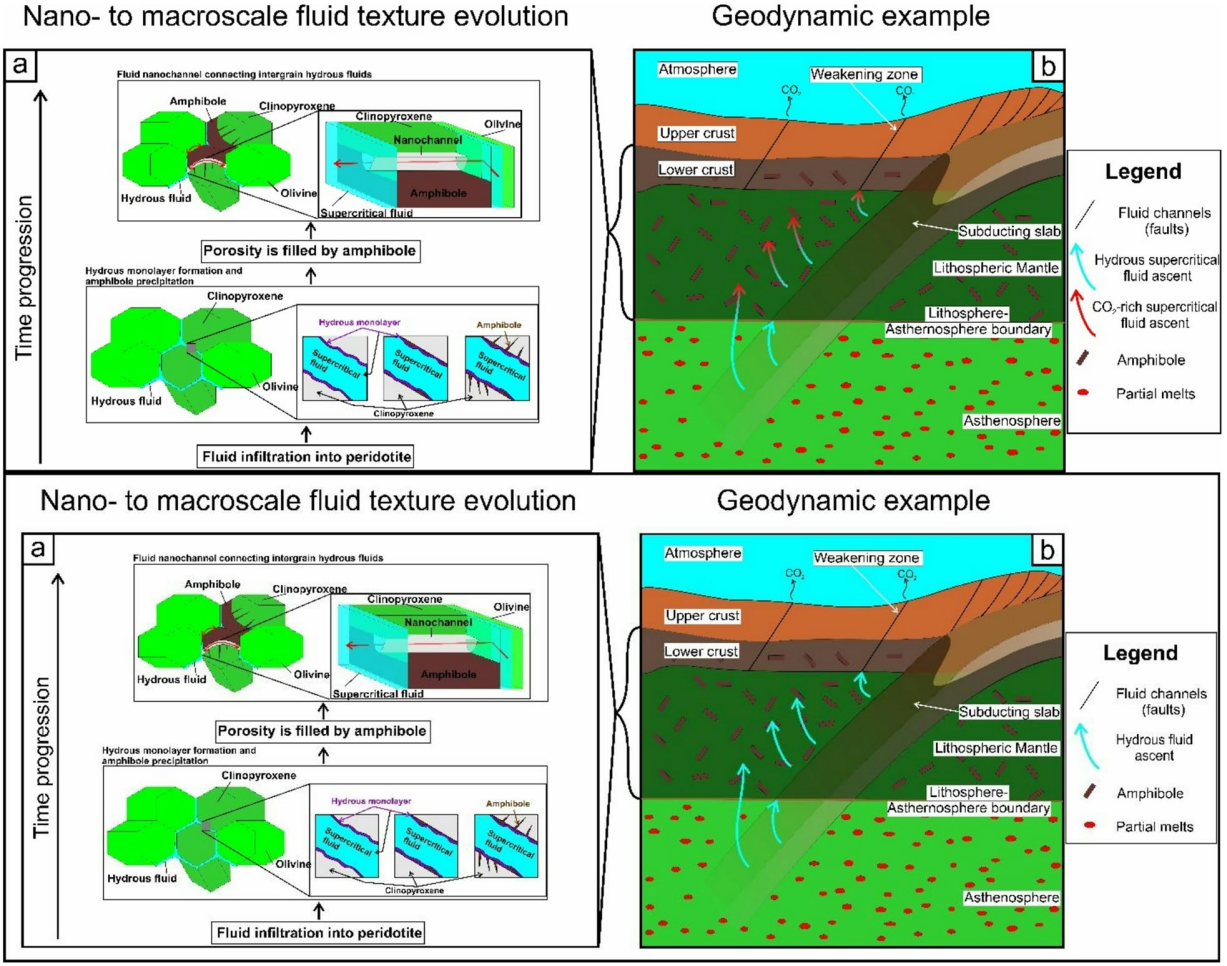
(**a**) Schematic figure of the progression of porosity decrease due to the formation of interstitial amphibole from clinopyroxene. Hydrous monolayers can be present at supercritical fluid-silicate interfaces as a result of H_2_O-silicate surface interaction^39–41^. Fluid flux transforms from fluid flow to pipe diffusion at the clinopyroxene-amphibole interface (red arrow) via nanochannels when porosity ceases. (**b**) Geodynamical setting of the process shown in (**a**), with the region of amphibole stability marked in darker shade. Due to amphibole formation the relative CO_2_ content of the supercritical fluid increases, and the fluid leaves the deep region along sub-vertical lithospheric-scale weakening zones.

The negative crystal shape of the volatile-rich mantle fluid inclusion (Fig. 1) results from a maturation process that tends to reduce the number of free surface bonds, thereby minimizing both the surface energy and the area of the solid-supercritical fluid interface (^17^; Supplementary Material; Fig. S6). This maturation takes place via dissolution-reprecipitation of the host clinopyroxene in the presence of CO_2_-H_2_O supercritical fluid under lithospheric mantle conditions^30^. During dissolution-reprecipitation process, it is likely that the H_2_O concentrates at the fluid inclusion walls (Fig. 4), and contains the dissolved silicate components of the host clinopyroxene that are present in the form of hydrous fluid complexes (e.g., NaAl(OH)_4_, Si(OH)_4_)^18^. This behaviour, and thus the higher bonding/adsorption affinity of H_2_O than CO_2_, is owed to the stronger polarity of the O-H bond (of H_2_O and hydrated complexes) compared to the O-C bond (of CO_2_)^37^; Fig. 4). In addition, the H_2_O Raman spectrum of Lange et al.^30^ suggests a small amount of dissolved NaCl in the entrapped H_2_O-bearing fluid^38^ that further enhances fluid surface wetting and silicate dissolution-reprecipitation^39^. Consequently, it assumes the differentiation of hydrous fluid species and CO_2_ at the solid-supercritical fluid interface for all fluid inclusions in the studied mantle xenolith, even though the entrapped supercritical fluid is always CO_2_-rich, as supported by the mass balance calculation for the studied upper mantle xenolith (Fig. 1; more details in Supplementary Material). As a result, few-nanometre-thick hydrous monolayers form at the clinopyroxene-supercritical fluid interface that are enriched in H_2_O (Fig. 4) and OH-bearing fluid complexes with respect to the bulk supercritical fluid^6^. The nature of the hydrous monolayers along the clinopyroxene-fluid interface at upper mantle conditions (Fig. 4) differ from those observed at surface and near-surface conditions^1^, attributable to the different physical properties (such as molecular density) between near-surface liquids and supercritical lithospheric mantle fluids^15,16^. The separation of fluid species can have a strong effect on CO_2_ abundancy within the hydrous monolayer, either as an individual molecule or in the form of [CO_3_]^2−^ species, such as even HCO_3_^−^^5^. Nevertheless, the enrichment of hydrated fluid complexes within a few nanometres distance from the fluid-clinopyroxene interface will lead to a continuous chemical gradient from the hydrated monolayer towards the bulk supercritical fluid (Fig. 4). The chemical equilibrium between the entrapped bulk supercritical fluid and the hydrous monolayer controls the abundancy of both ad- or absorbed hydrated fluid complexes^31^. Therefore, monolayers, such as the hydrated monolayer in Fig. 4, can create more saturated fluid conditions compared to the bulk supercritical fluid^9^. Eventually, the joint composition of the outermost clinopyroxene layer and the hydrated monolayer approaches an amphibole-like composition, if the hydrous monolayer also contains hydrous fluid complexes and, such as NaAl(OH)_4_, Si(OH)_4_ and individual ions, like Na^+^ (Fig. 4). Extrapolating Fig. 4 onto interstitial areas, it can be assumed that similar monolayers are likely present at interfaces between silicate minerals (e.g., olivine, clinopyroxene) and H_2_O-bearing, CO_2_-rich supercritical fluid within the lithosphere (Fig. 5), where the porosity and abundance of the supercritical fluid allow for it, thus offering rapid pathways to metasomatic reactions.

The slightly deformed texture of the studied upper mantle xenolith (Fig. 1) suggests a continuous stress, likely linked to the young geodynamic evolution of the lithospheric mantle beneath the Southeastern Carpathians^42,^ to which the rock-forming minerals react via structural rearrangement^42^. Supercritical fluid entrapment via fluid overpressure fracture formation and consequent fracture healing leads to a large number of structural defects (such as fracture dislocations^43^, that can migrate due to the external stress^44^. Relaxation of the crystal defects, including those that formed during fracture healing, will eventually lead to surface distortion (Fig. 4a**/II**). To maintain the negative crystal shape, dissolution of clinopyroxene starts where the dissolved material incorporates into the hydrous monolayer. This will lead to oversaturation of the presumably already saturated hydrous monolayer that, due to the amphibole-like composition of the solid-supercritical interface, will favour amphibole formation (Fig. 4a, b). This causes the incorporation of hydrous fluid complexes (like Na-Al[OH_4_]) present within the hydrous monolayers into the newly formed amphibole structure (Fig. 4a, b). As amphibole forms via the interaction of the hydrous monolayer and host clinopyroxene, it can be assumed that the hydrated fluid complexes play a role in the chemical reaction. Here, hydrated fluid complexes that contain Na and Al (and individual ions, such as Na^+^), based on the hydrous nano-silicate melt inclusion^30^; play an essential role in the amphibole-forming reaction as the studied amphibole is enriched in these elements relative to the host clinopyroxene^30^. Within the supercritical fluid, all listed ions and hydrated fluid complexes are carried by H_2_O molecules solvent^8,45^, suggesting that the transported molecules have a strong affinity to get incorporated into the solid than remaining in the solvent (i.e., H_2_O). Therefore, we approach the problem by incorporating known solutes into the amphibole-forming reaction along with the host clinopyroxene and the reaction product amphibole. Thus, by considering the assumed chemical compositions of the hydrated fluid complexes^18,46^, the most common end-members of the studied clinopyroxene and amphibole (diopside and pargasite, respectively^30^, the number of formula units in clinopyroxene and amphibole unit cells (Z = 4 and 2, respectively), and the replacement of 2 clinopyroxene by 1 amphibole unit cell, the following reaction can be assumed for the above fluid-mediated dissolution-reprecipitation process:

1 $$\begin{gathered} {\text{8 CaMg}}\left[ {{\mathrm{Si}}_{{\mathrm{2}}} {\mathrm{O}}_{{\mathrm{6}}} } \right]{\text{ }}\left( {{\mathrm{solid}}} \right)\, + \,{\text{2 NaAl}}\left( {{\mathrm{OH}}} \right)_{{\mathrm{4}}} \left( {{\mathrm{fluid}}} \right)\, + \,{\text{4 H}}_{{\mathrm{5}}} {\mathrm{AlSiO}}_{{\mathrm{6}}} \left( {{\mathrm{fluid}}} \right)\, \hfill \\ \quad = \,{\text{2 NaCa}}_{{\mathrm{2}}} \left( {{\mathrm{Mg}}_{{\mathrm{4}}} {\mathrm{Al}}} \right)\left[ {{\mathrm{Si}}_{{\mathrm{6}}} {\mathrm{Al}}_{{\mathrm{2}}} {\mathrm{O}}_{{{\mathrm{22}}}} } \right]\left( {{\mathrm{OH}}} \right)_{{\mathrm{2}}} \left( {{\mathrm{solid}}} \right)\, + \,{\text{4 H}}_{{\mathrm{6}}} {\mathrm{Si}}_{{\mathrm{2}}} {\mathrm{O}}_{{\mathrm{7}}} \left( {{\mathrm{fluid}}} \right)\, + \,{\text{4 CaO }}\left( {{\mathrm{fluid}}} \right). \hfill \\ \end{gathered}$$

In Eq. 1, the Na and Al of NaAl(OH)_4_ are incorporated from the solute into the amphibole A and C positions, respectively, whereas 2 (OH)^−^ is incorporated into the anion position. The residual 2 (OH)^−^ of the former NaAl-fluid complex contribute to the (Si[OH])^3+^ ↔ Al^3+^ exchange in the T (tetrahedron) position of amphibole, providing an opportunity for Si to get dissolved into the supercritical fluid. Note that Ca^2+^ solubility in volatile-rich supercritical fluid strongly depends on the presence and molecule speciation of the dissolved silicate melt components or anions (such as Cl^−^ or HCO_3_^−^)^47–49^ of which both components are present in the studied fluid inclusions^30^. The addition of Ca and Si to the newly formed fluid assumes that the process might enhance further Ca-silicate mineral (amphibole, clinopyroxene) formation or growth for environments of low Ca and Si. Besides solid solutions, impurities, such as H^40^, can also contribute to amphibole formation^50^, especially, in the vicinity of the secondary fluid inclusions (Fig. 1), where increased structural hydrogen content can be present.

The incorporation of more complex fluid species, such as larger silicate species^46^, into Eq. (1) provides an opportunity to expand Eq. (1) from a CO_2_-rich supercritical fluid towards higher silicate-content supercritical fluids. As amphibole formation, via clinopyroxene-supercritical fluid interaction, expands over large pressures and temperatures (up to 3 GPa and 1050 °C) and fluid compositional ranges^21–24^, various equations derived from Eq. (1) can be written for these systems with the incorporation of silicate melt components, such as K, Si, Al, Ti. The incorporation of CO_2_ into the left side of Eq. (1) provides an opportunity for carbonate formation if CO_2_ interacts with residual (OH) or oxides (CaO, MgO). If such reactions take place in regions where amphibole and carbonate are simultaneously stable^23^, it can be assumed that amphibole formation can have a significant effect on carbonate formation in the lithospheric mantle. Carbonate minerals observed in the fluid inclusions of the studied xenolith, however, cannot be used for confirming this hypothesis as they are highly likely late-stage solid phases that formed during xenolith transportation towards surface.

The transformation from a clinopyroxene + fluid two-phase system into a clinopyroxene + fluid + amphibole three-phase system likely occurs in the presence of amorphous silica such as the one observed by Konrad-Schmolke et al.^51^. The new three-phase system initiates chemical communication between the hydrated monolayers and the clinopyroxene-amphibole interface, where fluid diffuses from the former into the latter (Fig. 4c, d).

### Formation of nanochannels

The nanochannels are limited to the clinopyroxene-amphibole interface, assuming they have formed after nanoscale amphibole formation (Fig. 4). During amphibole growth, specifically the 6c-3a phase transformation (Figs. 2 and 3), the silicate tetrahedral chains combine (single to double) and the I-beams become wider. The misfit between the clinopyroxene and amphibole structures creates a nanochannel, aligned along the [001] direction^52^. The boundary of the misfit can be characterized by high surface potential and dielectric permittivity, representing the fastest diffusion pathway along the clinopyroxene-amphibole interface in the form of pipe diffusion^52^. Hydrogen bonds with surface oxygen via OH-bonds, along the surface of the nanochannels^53^, enhancing the diffusion of elements necessary for amphibole growth (e.g., Na, Al^27^). The enrichment of these elements is supported by the presence of nano-silicate melt inclusions along the clinopyroxene-amphibole interface^30^.

The presence of clinojimthompsonite at the 6c-3a interface (Fig. 2) is likely due to surface and stress minimization around the nanochannels, with this mineral being a transitional phase in the clinopyroxene to amphibole transformation^54^. Based on Thompson^54^, the compositions of multiple-chain silicates can be described by adding the pyroxene and mica modules (P and M, respectively) in their structures. This gives an opportunity to predict the composition of the three-chain biopyribole (Fig. 2). Using the diopside (P) and pargasite end-member compositions^30^ and the PMMP sequence of clinojimthompsonite^36^, we obtain compositions of Na(Mg_2_Al)[Si_2_Al_2_O_10_](OH)_2_ and Na_2_Ca_2_(Mg_6_Al_2_)[Si_8_Al_4_O_32_](OH)_4_ for the formulae of ‘M’ and clinojimthompsonite, respectively (Supplementary Material). Thus, the clinopyroxene interacts with the incompatible elements of the supercritical fluid (e.g., H, Na, Al) found mostly along the clinopyroxene-amphibole interface^30^ to form biopyribole. Eventually, clinojimthompsonite interacts with another clinopyroxene I-beam to form 2 amphibole I-beams (Supplementary Material). The clinojimthompsonite to amphibole transformation occurs by asymmetric edge dislocation migration^26^. This results in the rotation and translation of the silicate structure during which the clinojimthompsonite and clinopyroxene I-beams merge, and, due to the interfacial stress, the pyribole breaks apart and by merging with a pyroxene chain and then forms two amphibole I-beams. The composition of the two amphibole I-beams is equivalent to the chemical composition of clinojimthompsonite + clinopyroxene, supporting the described phase transformation mechanism (Supplementary Material). This process explains how a nanochannel termination and consequently opens/lengthens another neighbouring nanochannel. Thus, a constant pathway is provided from the fluid inclusion to the boundary of the amphibole lamella. The volatile-rich supercritical fluid and amphibole H_2_O mol% calculations (Supplementary Material) suggest that the volume of the newly-formed amphibole (Fig. 1/c) and the growth maintenance of the nanochannels (Fig. 2) strongly depend on the initial volume of the volatile-rich fluid inclusion and the H_2_O mol% of the entrapped supercritical fluid. Additionally, for the 2c-1a and 10c-5a even/odd interfaces (Supplementary Material) a different mechanism should be considered due to the absence of the pyribole and rare occurrence of these interfaces. Despite these facts, the presence of the empty spaces at these interfaces implies a similarly enhanced element diffusion compared to the even/even interfaces.

### Global geochemical implications

Amphibole formation and growth in the upper mantle, in the presence of hydrous supercritical fluid and surface monolayers, can occur ubiquitously in deep lithospheric, regional-scale processes and horizons^23^. Hydrous monolayers form after hydrous fluid infiltration along surfaces and induce dissolution-reprecipitation, leading to the maturation of the fluid-solid interface. Although supercritical fluid is thought to be homogeneous if bulk fluid is considered as a result of lithospheric mantle pressure and temperature environment, the hydrous monolayers suggest inhomogeneity along the solid-fluid interface (Fig. 4). Therefore, surface wetting is strongly controlled by the bulk CO_2_/H_2_O ratio, which is in agreement with the experimental observation of Watson and Brennan^40^ in terms of pore fluid geometry. In the deep lithosphere, supercritical fluid migrates via crack formation or diffusion along grain boundaries^11^. The CO_2_-rich supercritical fluids have high dihedral angle^55^ and need hydration or stress to initiate fluid migration^55,56^. The decrease of the dehydration angle results in the connectivity of the pore fluid enhancing element migration^55^. Furthermore, such fluid component as hydrogen can be stored to some extent as structural hydroxyl in nominally anhydrous mantle minerals^40^. In case of a very small amount ( < < 1 vol%.) of intergranular fluid, the consumption of fluid components has a high impact on the overall fluid quality. This causes rapid increase in the CO_2_ concentration and the dihedral angle of the residual fluid. Here, nanochannels present a pathway for CO_2_ to migrate along interfaces, in a clinopyroxene- and amphibole-rich environment where porosity is almost zero and only diffusion is possible (Fig. 5). As nanochannels have an almost constant width as they are crystallographic constrained (Fig. 2) they are not affected by the change of dihedral angle observed for the micron-scale fluids. In highly deformed zones in the lithosphere where amphibole formation occurs, clinopyroxene and amphibole can have a very strongly preferred crystal orientation^42^, and nanochannels along the clinopyroxene-amphibole interfaces are suggested to be parallel to the plane of the deep-seated weakening zones. Here, grain boundary wetting by the supercritical fluid will decrease due to amphibole growth (i.e., the relative amount of H_2_O decreases^55^); that can hinder fluid migration. Stress-induced fluid migration of CO_2_-rich supercritical fluids can be induced if the geodynamic environment is sufficiently active^56^. For clinopyroxene- and amphibole-rich deformation zones, the lack of fluid migration can be countered if nanochannels are present providing migration routes for H_2_O bearing CO_2_-rich supercritical fluids (Fig. 5). Thus, for these systems, the CO_2_ can be transported along deep-seated weakening zones towards shallower regions reaching the lower crust, contributing to the well-known lithosphere-scale fluid transport processes such as and mantle degassing (Fig. 5, e.g.,^57^).

Furthermore, regions where the nanochannel pathways can have a larger role in fluid migration are amphibole-rich zones in the lower crust (e.g., metamorphic rehydration processes^2^, and the upper mantle (e.g., the lithosphere-asthenosphere boundary (≤ 100 km)), and the supposed hydrous mid-lithospheric discontinuity^12^. Here, the consumption of H_2_O and associated supercritical fluid complexes (e.g., NaAl(OH)_4_) leads to a high amount of residual CO_2_ in the upper lithospheric mantle supercritical fluid (Supplementary Material) that spontaneously migrates towards the surface and contributes to the non-volcanic global geological CO_2_-rich emanation^12^ and references therein). The presence of other hydrous mantle minerals (e.g., phlogopite^58^) can increase the depth range of the above-mentioned process towards higher pressure domains. These results provide insight into the nano- to lithospheric-scale fluid composition evolution and raise new questions that warrant further nanoscale experimental and modelling studies (e.g.,^59^).

## Supplementary Information

Below is the link to the electronic supplementary material.

Supplementary Material 1

## Data Availability

Data are provided within the manuscript or supplementary information file.
