# Supplementary material for: Nanoscale solid-fluid interaction and amphibole formation in the lithospheric mantle
Source: Sci Rep. 2026 Feb 17;16:11009. doi: 10.1038/s41598-026-40179-1 (PMC13043934; doi:10.1038/s41598-026-40179-1)
Supplement: Supplementary file 1 — Supplementary Material 1 [file 41598_2026_40179_MOESM1_ESM.docx]

**Supplementary**

1. **Geological background**

The Perşani Mountains Volcanic Field (PMVF), located at the boundary of the Transylvanian Basin and Southeastern Carpathians, is the southeasternmost and youngest (1.2-0.6 Ma, [1]) Neogene alkaline monogenetic volcanic field within the Carpathian-Pannonian region (CPR), Central Europe (**Fig. 1 in main text**). The volcanism was simultaneously active with the calc-alkaline volcanism of the Harghita Mountains ([2]; [3]) in a compressional tectonic regime after the collision of the Tisza-Dacia microcontinent and the Moesian and European platforms ([4]). Six volcanic periods are distinguished based on K-Ar, Ar-Ar chronometry and field observations ([1]; [5]) forming eruption centers along a northeast–southwest and northwest–southeast directions that aligns with the regional major tectonic structures ([5]). The PMVF lies approximately 50 km northwest to the Vrancea seismogenic zone, which is known by the occurring high (>6 Mw) magnitude intermediate-depth earthquakes initiated by the actively sinking Vrancea slab (e.g., [6]; [7]; [8]). In the hinterland, an asthenospheric upwelling ([9]) initiated the PMVF alkaline basalt volcanism ([10]; [11]). Abundant mantle xenoliths were sampled from the uplifted mantle wedge by the basalt magma and are hosted by tuffs and lavas flows ([12]; [13]; [14]). The abundance of amphibole ([12]), major and trace element composition of pyroxenes and amphiboles in mantle xenoliths ([14]), combined with trace element composition of the alkaline basalts ([15]) support an enriched lithospheric mantle wedge underneath the PMVF that suffered intensive deformation based on the presence of mylonitic xenoliths ([13]).

**Petrography**

The studied samples (PGR-X1-0345) is an amphibole-bearing lherzolite xenolith with a modal composition of Ol_55_Opx_20_Cpx_16_Sp_5_Amph_4_. The xenolith has an oriented, proto-porphyroclastic texture, where spinel distribution defines the xenolith foliation. Porphyroclasts are orthopyroxene, rarely clinopyroxene. Olivine forms anhedral grains with a grain size range of 1000 to 3500 µm and shows subgrains and kink bands deformation signatures. Orthopyroxene is anhedral and forms two size ranges (< 1000 µm and 2500 µm <), from which the larger grains are the porphyroclasts, whereas the smaller grains form bands between olivine grains that are parallel to the foliation. Large orthopyroxene crystals show deformation patterns such as deformation lamellae and bending often associated with clinopyroxene and spinel exsolution lamellae. In addition, amphibole overgrowths occur in places around the orthopyroxene porphyroclasts. Clinopyroxene is anhedral, showing a continuous distribution of grain sizes from 500 to 2500 µm and displays no sign of deformation, and occurs dominantly in the same textural relation as the smaller orthopyroxenes. Large clinopyroxenes contain orthopyroxene and amphibole exsolution lamellae. Spinel forms anhedral, elongated crystals or occurs as exsolution lamellae in orthopyroxene. Length of the elongated spinel crystals ranges between 100-2000 µm. Amphiboles can be categorized as interstitial, lamella or fluid associated amphibole. Interstitial amphibole form tabular subhedral crystals and are located in the vicinity to porphyroclastic pyroxenes and spinel grains. Their size ranges between 100 to 1000 µm and show no signs of deformation. Amphibole exsolution lamellae form elongated and thin phases in clinopyroxene. In some cases, amphibole exsolution can reach the host clinopyroxene grain boundary and be connected to interstitial amphiboles. These exsolution lamellae are not related to fluid inclusions. In addition, clinopyroxene sometimes hosts amphiboles that are always associated with fluid inclusions. These amphiboles, the main subject of this study, are always in physical contact with the fluid inclusions and are referred to as fluid inclusion associated amphibole lamellae (s.s. [16]). They form tabular crystals that are typically oriented parallel to each other. Fluid inclusion associated amphibole lamellae are absent in orthopyroxene, where only spinel and clinopyroxene lamellae are present.

**References**

[1] Panaiotu, C. G., et al. ^40^Ar/^39^Ar chronology and paleomagnetism of Quaternary basaltic lavas from the Perşani Mountains (East Carpathians). *Physics of the Earth and Planetary Interiors*, **221**, 1-14. <https://doi.org/10.1016/j.pepi.2013.06.007> (2013).

[2] Pécskay, Z., Lexa, J., & Kovács, M. (1995). Space and time distribution of Neogene-Quaternary volcanism in the Carpatho-Pannonian region. *Acta Vulcanologica*, **7(2)**, 15-28.

[3] Molnár, K., et al. Noble gas geochemistry of phenocrysts from the Ciomadul volcanic dome field (Eastern Carpathians). *Lithos*, **394**, 106152. <https://doi.org/10.1016/j.lithos.2021.106152> (2021).

[4] Mațenco, L., et al. Large‐scale deformation in a locked collisional boundary: Interplay between subsidence and uplift, intraplate stress, and inherited lithospheric structure in the late stage of the SE Carpathians evolution. *Tectonics*, **26(4)**. <https://doi.org/10.1029/2006TC001951> (2007).

[5] Seghedi, I., Popa, R. G., Panaiotu, C. G., Szakács, A., & Pécskay, Z. Short-lived eruptive episodes during the construction of a Na-alkalic basaltic field (Perşani Mountains, SE Transylvania, Romania). *Bulletin of Volcanology*, **78**, 1-16. <https://doi.org/10.1007/s00445-016-1063-y> (2016).

[6] Knapp, J. H., et al. Crustal constraints on the origin of mantle seismicity in the Vrancea Zone, Romania: The case for active continental lithospheric delamination. *Tectonophysics*, **410(1-4)**, 311-323. <https://doi.org/10.1016/j.tecto.2005.02.020> (2005).

[7] Tondi, R., Achauer, U., Landes, M., Davi, R., & Besutiu, L. Unveiling seismic and density structure beneath the Vrancea seismogenic zone, Romania. *Journal of Geophysical Research: Solid Earth*, **114(B11)**. <https://doi.org/10.1029/2008JB005992> (2009).

[8] Kovács, I. J., et al. The ‘pargasosphere’ hypothesis: Looking at global plate tectonics from a new perspective. *Global and Planetary Change*, **204**, 103547. <https://doi.org/10.1016/j.gloplacha.2021.103547> (2021).

[9] Falus, G., Szabó, C., & Vaselli, O. Mantle upwelling within the Pannonian Basin: evidence from xenolith lithology and mineral chemistry. Terra nova, **12(6)**, 295-302. <https://doi.org/10.1046/j.1365-3121.2000.00313.x> (2000).

[10] Harangi, S., Sági, T., Seghedi, I., & Ntaflos, T. (2013). Origin of basaltic magmas of Perşani volcanic field, Romania: A combined whole rock and mineral scale investigation. *Lithos*, **180**, 43-57. <https://doi.org/10.1016/j.lithos.2013.08.025>

[11] Bracco Gartner, A. J., Seghedi, I., Nikogosian, I. K., & Mason, P. R. (2020). Asthenosphere-induced melting of diverse source regions for East Carpathian post-collisional volcanism. *Contributions to Mineralogy and Petrology*, **175(6)**, 54. <https://doi.org/10.1007/s00410-020-01690-4>

[12] Vaselli, O., Downes, H., Thirlwall, M., Dobosi, G., Coradossi, N., Seghedi, I., Szakács, A., & Vannucci, R. (1995). Ultramafic xenoliths in Plio-Pleistocene alkali basalts from the Eastern Transylvanian Basin: depleted mantle enriched by vein metasomatism. *Journal of Petrology*, **36(1),** 23-53. <https://doi.org/10.1093/petrology/36.1.23>

[13] Falus, G., Tommasi, A., & Soustelle, V. (2011). The effect of dynamic recrystallization on olivine crystal preferred orientations in mantle xenoliths deformed under varied stress conditions. *Journal of Structural Geology*, **33(11)**, 1528-1540. <https://doi.org/10.1016/j.jsg.2011.09.010>

[14] Faccini, B., Rizzo, A. L., Bonadiman, C., Ntaflos, T., Seghedi, I., Grégoire, M., Ferretti, G., & Coltorti, M. (2020). Subduction-related melt refertilisation and alkaline metasomatism in the Eastern Transylvanian Basin lithospheric mantle: Evidence from mineral chemistry and noble gases in fluid inclusions. *Lithos*, **364**, 105516. <https://doi.org/10.1016/j.lithos.2020.105516>

[15] Downes, H., Seghedi, I., Szakacs, A., Dobosi, G., James, D. E., Vaselli, O., Rigby, I. J., Ingram, G. A., Rex, D., & Pécskay, Z. (1995). Petrology and geochemistry of late Tertiary/Quaternary mafic alkaline volcanism in Romania. *Lithos*, **35(1-2)**, 65-81. <https://doi.org/10.1093/petrology/42.1.233>

[16] Lange, T. P., Pálos, Z., Pósfai, M., Berkesi, M., Pekker, P., Szabó, Á., Szabó, Cs., & Kovács, I. J. (2023). Nanoscale hydrous silicate melt inclusions at the clinopyroxene-amphibole interface in a mantle xenolith from the Perșani Mountains Volcanic Field. *Lithos*, **454**, 107210. <https://doi.org/10.1016/j.lithos.2023.107210>

**2. Sampling and Methods**

The studied xenolith (PGR-X1-0345) originates from Gruiu (45°57’19.4”N 25°20’05.7”E; Supplementary 1), located in the central part of the Perșani Mountains Volcanic Field. From the xenolith, we prepared doubly polished thick section (~150 µm) and used for petrographic observation a Nikon Eclipse LV100 POL polarizing microscope at the Lithosphere Fluid Research Laboratory (LRG) at the Department of Petrology and Geochemistry, Eötvös Loránd University (Hungary).

*Focused Ion Beam-Scanning Electron Microscopy*

In the studied xenolith, textural relation between the host clinopyroxene and amphibole tabular intergrowth was revealed by the Focused Ion Beam-Scanning Electron Microscope (FIB-SEM) technique using a FEI Quanta 3D dual-beam scanning electron microscope with both secondary and backscattered electron detectors at the ELTE-FS-RICF. This was followed by the preparation of multiple approximately 100 nm thick TEM lamella oriented perpendicular to the longitudinal axis (c crystallographic axis) of the amphibole intergrowth. During preparation a Pt coating was deposited on the section of interest, followed by a two-sided milling with a Ga ion laser. The prepared TEM lamella was finally placed on a Cu TEM lamella holder.

*Transmission Electron Microscopy*

Transmission Electron Microscopy (TEM) analyses were performed at the Nanolab at the University of Pannonia (Hungary) using a Talos F200X G2 instrument (Thermo Fisher), operated at 200 kV accelerating voltage, equipped with a field-emission gun and a four-detector Super-X energy-dispersive X-ray spectrometer, capable of working in both conventional TEM and scanning transmission (STEM) modes. Low-magnification bright-field (BF) images, high-resolution (HRTEM) images and selected-area electron diffraction (SAED) patterns were obtained in TEM mode. The scanning transmission electron microscopy high-angle annular dark-field (STEM HAADF) images were collected for both high-resolution structure analyses and for mapping elemental compositions by coupling STEM imaging with energy-dispersive X-ray spectrometry (EDS). For a detailed description regarding HAADF imaging of silicate minerals, the reader is referred to the work of Rodriguez et al ([1]).

**References**

[1] Rodriguez, Y. T. C., Ciobanu, C. L., Slattery, A., Cook, N. J., Schutesky, M. E., Ehrig, K., King, S. A., & Yao, J. (2024). Polysomatic intergrowths between amphiboles and non-classical pyriboles in magnetite: smallest-scale features recording a protracted geological history. *American Mineralogist*.

**3. Calculation the initial CO_2_ and H_2_O mol % of the original fluid inclusion**

The presence of H_2_O in fluid inclusion from mantle rocks was determined by in-situ Raman spectroscopy measurements (e.g., [1]; [2]) proven to be present in sufficient amount to produce amphibole (e.g., [3]). During amphibole formation and growth, the H_2_O content (and thus the molar H_2_O ratio) of the mantle fluid decreases and results in the relative enrichment of other – non-amphibole favoured – components (e.g., CO_2_, N_2_, H_2_S; e.g., [4]; [5]).

Similar process was observed in the studied slightly deformed lherzolite mantle xenolith (PGR-X1-0345), where the fluid composition changes during amphibole formation. The system resembles a quasi-closed system, compared to intergrain system, and therefore provides a possibility to better understand the fluid-amphibole compositional-volumetric relation. As the CO_2_ and H_2_O are the most abundant volatile phases within the studied fluid inclusions (and mantle fluids; [1]; [2]; [6]), we determined how much H_2_O loss can occur during amphibole formation from the original fluid inclusion in a supercritical CO_2_-H_2_O system. In addition, by determining the amount of newly formed amphibole the estimation of the minimum Na and Al of the entrapped fluid can also be considered.

**H_2_O content of the newly formed amphibole**

For the amphibole, H_2_O content calculation we hypothesized that the amphibole only contains (OH) in the anion position, which is in strong agreement with the compositions of Lange et al. ([5]). Then, we defined a 10-10000 μm^3^ mineral volume range and a 907.78 Å^3^ unit cell volume ([5]) for the amphibole. Each amphibole unit cell contains two amphibole molecules (Z = 2) ([7]), thus the amount hydrogen within a pure (OH)-amphibole unit cell is 4. By determining the amount of unit cells in a given mineral volume, the amount of hydrogens can be determined for the mineral volume from which the H_2_ molar quantity can be calculated. The given H_2_ molar quantity is equivalent to the amount of molar H_2_ found in the same amount of H_2_O.

**Table S1**. The H_2_O mol for four random amphiboles. The unit cell volume is from Lange et al. (2023). Amp = amphibole.

|  | Mineral volume (μm^3^) | Unit cell volume (Å^3^) | Mineral volume /unit cell volume | Number of hydrogen | H_2_O mol |
| --- | --- | --- | --- | --- | --- |
| Amp1 | 10 | 907.78 | 1.10*10^10^ | 4.41*10^10^ | 3.66*10^-14^ |
| Amp2 | 200 | 907.78 | 2.20*10^11^ | 8.81271*10^11^ | 7.32*10^-13^ |
| Amp3 | 5000 | 907.78 | 5.51*10^12^ | 2.20318*10^13^ | 1.83*10^-11^ |
| Amp4 | 10000 | 907.78 | 1.10*10^13^ | 4.40635*10^13^ | 3.66*10^-11^ |

**H_2_O content of the observed fluid inclusions**

Volatile-rich fluid inclusions in ortho- and clinopyroxene of mantle xenoliths from the Carpathian-Pannonian region have a CO_2_ molar content range of 85-100 mol% (e.g., [8]; [9]; [10]; [6]) providing a maximum range for H_2_O quantity when a pure CO_2_-H_2_O system is estimated). Furthermore, CO_2_ and H_2_O within fluid inclusions trapped under lithospheric mantle conditions have a density around 1 g/cm^3^ (e.g., [6, 8, 9]). To calculate the exact amount of H_2_O mol within the fluid inclusion at the μm^3^ scale, the following equation should be used:

$n_{1}= \frac{V*x_{1}* \rho}{M}$, where

n_1_ is the present H_2_O mol quantity of the fluid inclusion (observed with e.g., Raman spectroscopy), V the volume of the fluid inclusion in cm^3^ (converted from μm^3^), x_1_ is the molar proportion of present H_2_O within the fluid inclusion, ρ is the density of H_2_O within the fluid inclusion and M is the molar weight of H_2_O. For the calculations , the volume range of the fluid inclusion is defined from 10-5000 μm^3^.

Note: When other incompatible elements (e.g., N, S, Si, Al, Na) are present the calculated H_2_O content decreases. Therefore, when the volume of the fluid inclusion and the H_2_O mol% of the entrapped fluid are defined one can estimate the minimum initial H_2_O quantity within the fluid inclusion.

**Table S2**. The calculated amount of H_2_O mol for 10 and 5 mol% for 4 random fluid inclusions within the 10-5000 μm^3^ range. FI = fluid inclusion.

|  | FI volume (μm^3^) | H_2_O mol% of the entrapped fluid | H_2_O molecule in the entrapped fluid | H_2_O mol% of the entrapped fluid | H_2_O molecule in the entrapped fluid |
| --- | --- | --- | --- | --- | --- |
| FI1 | 10 | 10 | 5.56*10^-14^ | 5 | 2.77*10^-14^ |
| FI2 | 200 | 10 | 1.11*10^-12^ | 5 | 5.56*10^-13^ |
| FI3 | 1000 | 10 | 5.56*10^-12^ | 5 | 2.77*10^-12^ |
| FI4 | 5000 | 10 | 2.77*10^-11^ | 5 | 1.39*10^-10^ |

**Table S3**. The calculated amount of CO_2_ mol for 90 and 95 mol% for 4 random fluid inclusions within the 10-5000 μm^3^ range. FI = fluid inclusion.

|  | FI volume (μm^3^) | CO_2_ mol% of the entrapped fluid | CO_2_ molecule in the entrapped fluid | CO_2_ mol% of the entrapped fluid | CO_2_ molecule in the entrapped fluid |
| --- | --- | --- | --- | --- | --- |
| FI1 | 10 | 90 | 2.05*10^-13^ | 95 | 2.16*10^-13^ |
| FI2 | 200 | 90 | 4.09*10^-12^ | 95 | 4.32*10^-12^ |
| FI3 | 1000 | 90 | 2.05*10^-11^ | 95 | 2.16*10^-11^ |
| FI4 | 5000 | 90 | 1.02*10^-10^ | 95 | 1.08*10^-10^ |

**H_2_O molar content of the original fluid inclusions**

Table 1-2 show that the volume of the fluid inclusion, which hosts 5 mol% H_2_O, should exceed the volume of the newly formed amphibole. In contrast, fluid inclusion which hosts 10 mol% H_2_O has sufficient H_2_O to form at least amphibole that has the same volume.

Based on the above described calculation, fluid inclusion - amphibole pairs can be made based on petrographic observation. After pairing, the H_2_O mol content of amphibole can be added to the fluid inclusion which now hosts the total H_2_O content of both the fluid inclusion and adjacent amphibole. Thus, the new bulk composition of the fluid inclusion should be normalized to 100 %. This leads to an increased H_2_O molar content compared to the initial H_2_O molar content and the decrease of other volatile substances (e.g., CO_2_).

In case of a H_2_O-CO_2_ fluid system, the initial CO_2_ and H_2_O molar content can be calculated with the help of Table 1-3. For example, a pair of fluid inclusion with a volume of 1000 μm^3^ and 5 mol% H_2_O together with amphibole with a volume of 200 μm^3^ are considered. Based on Table 1-2, the measured total molar H_2_O of both phases is approximately equal to 3.502*10^-12^ mol, which is ~ 26% increase of the fluid H_2_O content. The molecule quantity of the bulk CO_2_-H_2_O fluid system corresponds to 2.51*10^-11^ mol that is 3 % more than the initial fluid 2.44*10^-11^ (= 2.16*10^-11^ +2.77*10^-12^) mol content. To determine the original H_2_O molar ratio, equation (1) should be rearranged to the unknown x:

$x_{2}= \frac{n_{2} * M}{V * \rho}$.

Here, n_2_ is equal to the new molar quantity of H_2_O (fluid inclusion + amphibole), whereas the molar weight (M), volume (V) and H_2_O density stayed the same. As n corresponds to 3.502*10^-12^ mol, the H_2_O molar ratio (x_2_) corresponds to 6.32 changing the initial 95 CO_2_ mol % to 93.68.

**Table S4**. Results of three calculation for three random fluid inclusion amphibole pairs. FI = fluid inclusion, amph = amphibole.

|  | Present H_2_O | Present CO_2_ | Amphibole volume | FI volume | Initial H_2_O | Initial CO_2_ |
| --- | --- | --- | --- | --- | --- | --- |
| FI+amph (1) | 5 | 95 | 50 | 600 | 5.55 | 94.45 |
| FI+amph (2) | 8 | 92 | 300 | 400 | 12.94 | 87.06 |
| FI+amph (3) | 12 | 88 | 500 | 4000 | 12.82 | 87.18 |

**References**

[1] Berkesi, M., Guzmics, T., Szabó, Cs., Dubessy, J., Bodnar, R. J., Hidas, K., & Ratter, K. The role of CO_2_-rich fluids in trace element transport and metasomatism in the lithospheric mantle beneath the Central Pannonian Basin, Hungary, based on fluid inclusions in mantle xenoliths. *Earth and Planetary Science Letters*, **331**, 8-20. <https://doi.org/10.1016/j.epsl.2012.03.012> (2012).

[2] Frezzotti, M. L., Ferrando, S., Tecce, F., & Castelli, D. Water content and nature of solutes in shallow-mantle fluids from fluid inclusions. *Earth and Planetary Science Letters*, **351**, 70-83. <https://doi.org/10.1016/j.epsl.2012.07.023> (2012a).

[3] Green, D. H. Experimental petrology of peridotites, including effects of water and carbon on melting in the Earth’s upper mantle. *Physics and Chemistry of Minerals*, **42**, 95-122. <https://doi.org/10.1007/s00269-014-0729-2> (2015).

[4] Kovács, I. J., et al. The ‘pargasosphere’ hypothesis: Looking at global plate tectonics from a new perspective. *Global and Planetary Change*, **204**, 103547. <https://doi.org/10.1016/j.gloplacha.2021.103547> (2021).

[5] Lange, T. P., et al. Nanoscale hydrous silicate melt inclusions at the clinopyroxene-amphibole interface in a mantle xenolith from the Perșani Mountains Volcanic Field. *Lithos*, **454**, 107210. <https://doi.org/10.1016/j.lithos.2023.107210> (2023).

[6] Aradi, L. E., et al. Geochemical evolution of the lithospheric mantle beneath the Styrian Basin (Western Pannonian Basin). *Lithos*, *378*, 105831. <https://doi.org/10.1016/j.lithos.2020.105831> (2020).

[7] Hawthorne, F. C., Oberti, R., Harlow, G. E., Maresch, W. V., Martin, R. F., Schumacher, J. C., & Welch, M. D. (2012). Nomenclature of the amphibole supergroup. *American Mineralogist*, **97(11-12)**, 2031-2048. <https://doi.org/10.2138/am.2012.4276>

[8] Hidas, K., et al. Coexisting silicate melt inclusions and H_2_O-bearing, CO_2_-rich fluid inclusions in mantle peridotite xenoliths from the Carpathian–Pannonian region (central Hungary). Chemical Geology, **274(1-2)**, 1-18 (2010). <https://doi.org/10.1016/j.chemgeo.2010.03.004>

[9] Berkesi, M., Czuppon, G., Szabó, Cs., Kovács, I., Ferrero, S., Boiron, M. C., & Peiffert, C. (2019). Pargasite in fluid inclusions of mantle xenoliths from northeast Australia (Mt. Quincan): evidence of interaction with asthenospheric fluid. *Chemical Geology*, **508**, 182-196. <https://doi.org/10.1016/j.chemgeo.2018.06.022>

[10] Créon, L., et al. Highly CO_2_-supersaturated melts in the Pannonian lithospheric mantle–A transient carbon reservoir?. *Lithos*, **286**, 519-533. <https://doi.org/10.1016/j.lithos.2016.12.009> (2017).

**4. Additional observations of the clinopyroxene-amphibole interfaces**

***(100) phase boundary****: Even-even clinopyroxene to amphibole transformation (s.s. [1])*

The even-even phase transformation results in a coherent (100) boundary between clinopyroxene and amphibole. The most common clinopyroxene-amphibole structural relation is where amphibole in the (100) direction ends with a ½ amphibole I beam (equivalent to 1 clinopyroxene I beam) (**Fig. 1** in main text). At the centre of the newly forming amphibole I beam, the I beam of the clinopyroxene is missing, but occasionally the gap is filled by an inclined clinopyroxene I beam. In addition, amphibole I beam is inclined in the [100] direction towards the clinopyroxene. The degree of amphibole I beam inclination is bigger when the clinopyroxene I beam is missing compared to when the gap is filled.

***(100) phase boundary****: Even-odd clinopyroxene to amphibole transformation (s.s. [1])*

The even-odd phase transformation is incoherent and forms an asymmetric ‘edge dislocation’ type structure (**Figure 1** **in main text**). The incoherency is observed in the form of missing amphibole I beam replaced by a ‘void’ at the centre of the asymmetric edge structure. Most common phase boundary occurs in the form of 6 clinopyroxene to 3 amphibole planes, although 2 clinopyroxene to 1 amphibole and 10 clinopyroxene to 5 amphibole plane transformations can also be observed. The clinopyroxene and amphibole structures surrounding the voids are banded that decreases in the (100) direction away from the ‘void’. When 3 new amphiboles form a third chain silicate phase, a pyribole, can also be observed (**Fig. 1 in the main text**). The pyribole has a length of 3 clinopyroxene/1.5 amphibole I beam in the [010] direction. The pyribole adjusts to the amphibole and clinopyroxene structure. The ‘void’ has a constant width from the clinopyroxene (010) end until the centre and from there continuously narrows down until the amphibole (010) end of the ‘void’. Additionally, the edge shape results in an extra clinopyroxene (100) plane than the amphibole. In some cases, tilting and bending can be observed at the amphibole structure that is found at the edge of the ‘void’ expanding the amphibole unit cell. In few cases the ‘void’ is filled by a clinopyroxene and amphibole octahedral I beam and associated silicate tetrahedrons (**Fig. 1 in the main text**). The inner clinopyroxene and amphibole I beams show a high degree of misorientation.

The second most abundant even/odd interface is the 2 clinopyroxene to 1 amphibole (2c-1a; **Fig. S2a**) interface that is associated with a smaller ‘empty’ area that in case of 6c-3a. In this case, clinojimthompsonite is absent. Therefore, less clinopyroxene and amphibole I beams inclination can be observed, although amphibole I beams adjacent to the empty space show high inclination compared to other 2c-1a clinopyroxene and amphibole I beams. The least abundant even/odd interface is the 10 clinopyroxene to 5 amphibole (10c-5a; **Fig. S2b**) interface that is associated with the largest ‘empty’ area of all observed even/odd clinopyroxene-amphibole interfaces. Similar to the 2c-1a interface the clinojimthompsonite is absent. The shape of the ‘empty’ space follows the clinopyroxene-amphibole interface forming a curved morphology in contrast to the 6c-3a and 2c-1a ‘empty’ areas. Therefore, amphibole and clinopyroxene I beams of the 10c-5a interface are almost parallel to the shape of the ‘empty’ area and are less inclined than the I beams observed at the 6c-3a and 2c-1a interface.


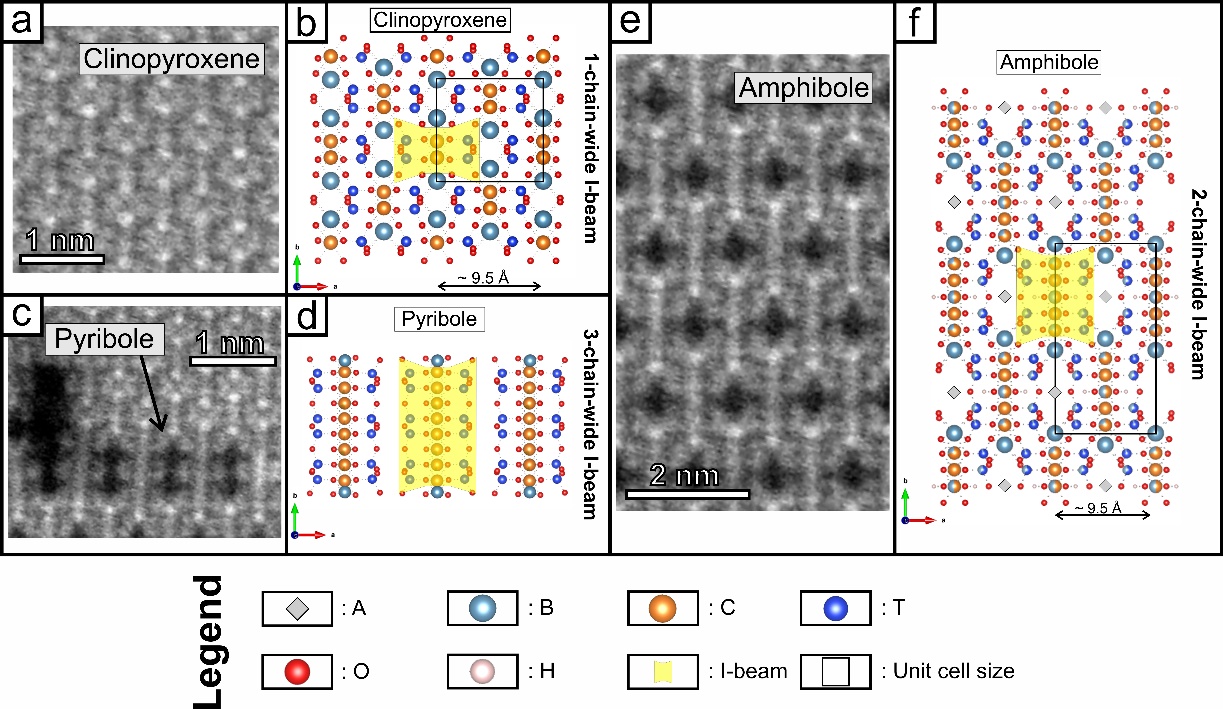


**Fig. S1.** High-angle annular dark field (HAADF) and atomic structure of clinopyroxene (a-b), pyribole (c-d) and amphibole (e-f).


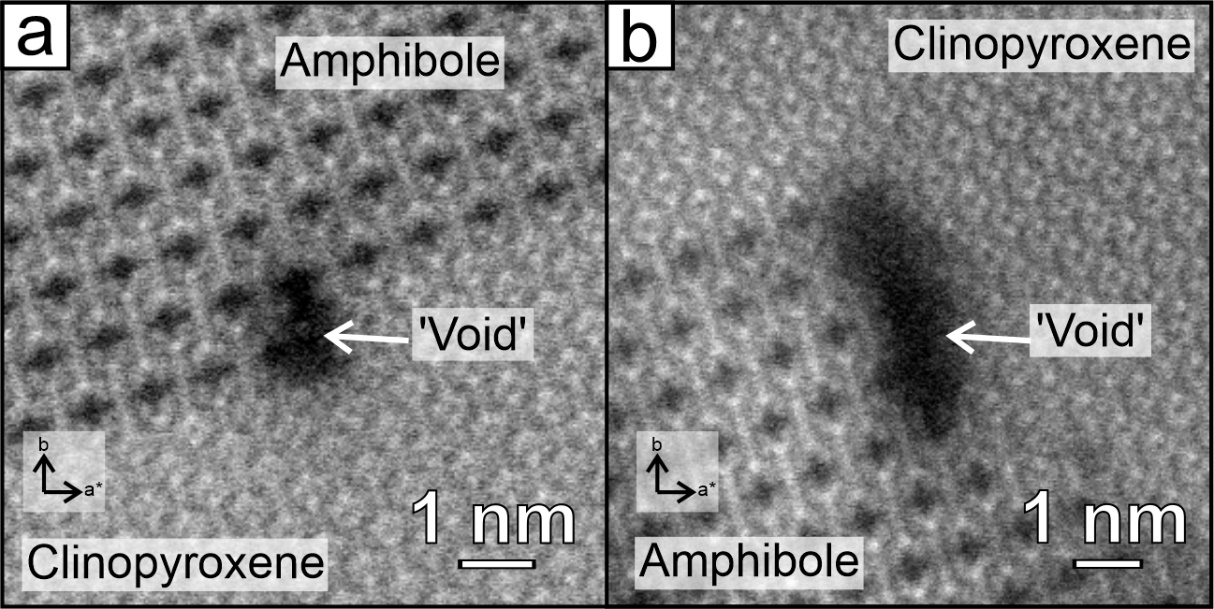


**Fig. S2.** High-angle annular dark-field (HAADF) images of the (**a**) 2 clinopyroxene to 1 amphibole and (**b**) 10 clinopyroxene to 5 amphibole I beam clinopyroxene-amphibole interfaces.

**Reference**

[1] Veblen, D. R., & Buseck, P. R. Hydrous pyriboles and sheet silicates in pyroxenes and uralites: intergrowth microstructures and reaction mechanisms. American Mineralogist, **66(11-12)**, 1107-1134 (1981).

**5. Virtual topology display of the nanochannel and surrounding area**


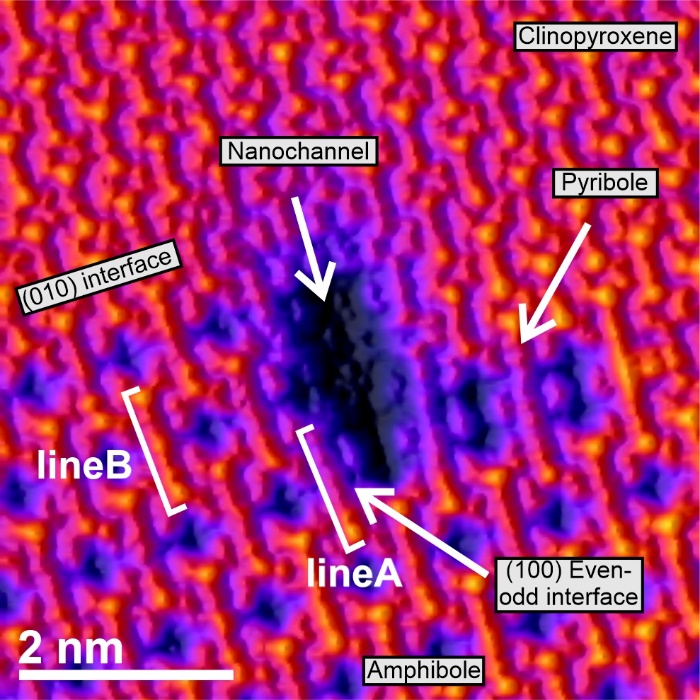


**Fig. S3.** False-colour, virtual topological image of a nanochannel and its surroundings calculated from the pixel intensities of an atomic-resolution scanning transmission electron microscopy high angular annular dark field (STEM HAADF) image. The virtual topology displays the magnitude of the HAADF signal and the bright yellowish colours and the dark purple and blue colours represent the higher and the lower HAADF signal intensities, respectively. The black areas are below the minimum value of the visualised intensity range. Since the HAADF signal depends both on the local thickness and the chemical composition (Z-contrast), the intensity alterations can be caused by either thickness changes and/or the compositional differences around the nanochannel. The applied colour rendering clearly shows that the octahedral positions bordering the nanochannel (line A) display lower intensity compared to similar positions further away from the channel (line B). A similar decrease of intensity is apparent at the tetrahedral positions. Since significant compositional changes in tetrahedral positions are not likely, we assume that the reduction of thickness contributes the decreased image intensity around the nanochannel; thus, the walls of the nanochannel are not apparently flat on the atomic scale through the entire sample thickness.


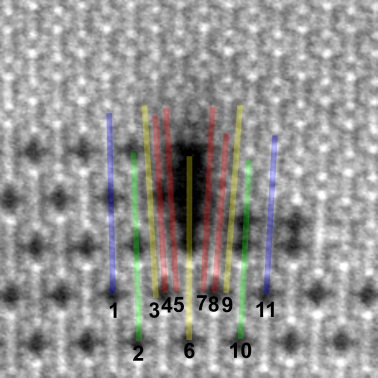


**Fig. S4.** The selected profiles along the intensity profiles are constructed and presented in **Supplementary Fig. S5**. The intensity profiles are parallel to the (100) even-odd interface following the amphibole (1-6), pyribole (7-11) and clinopyroxene (7-11) I-beams. Profile 6 penetrates the centre of the nanochannel.


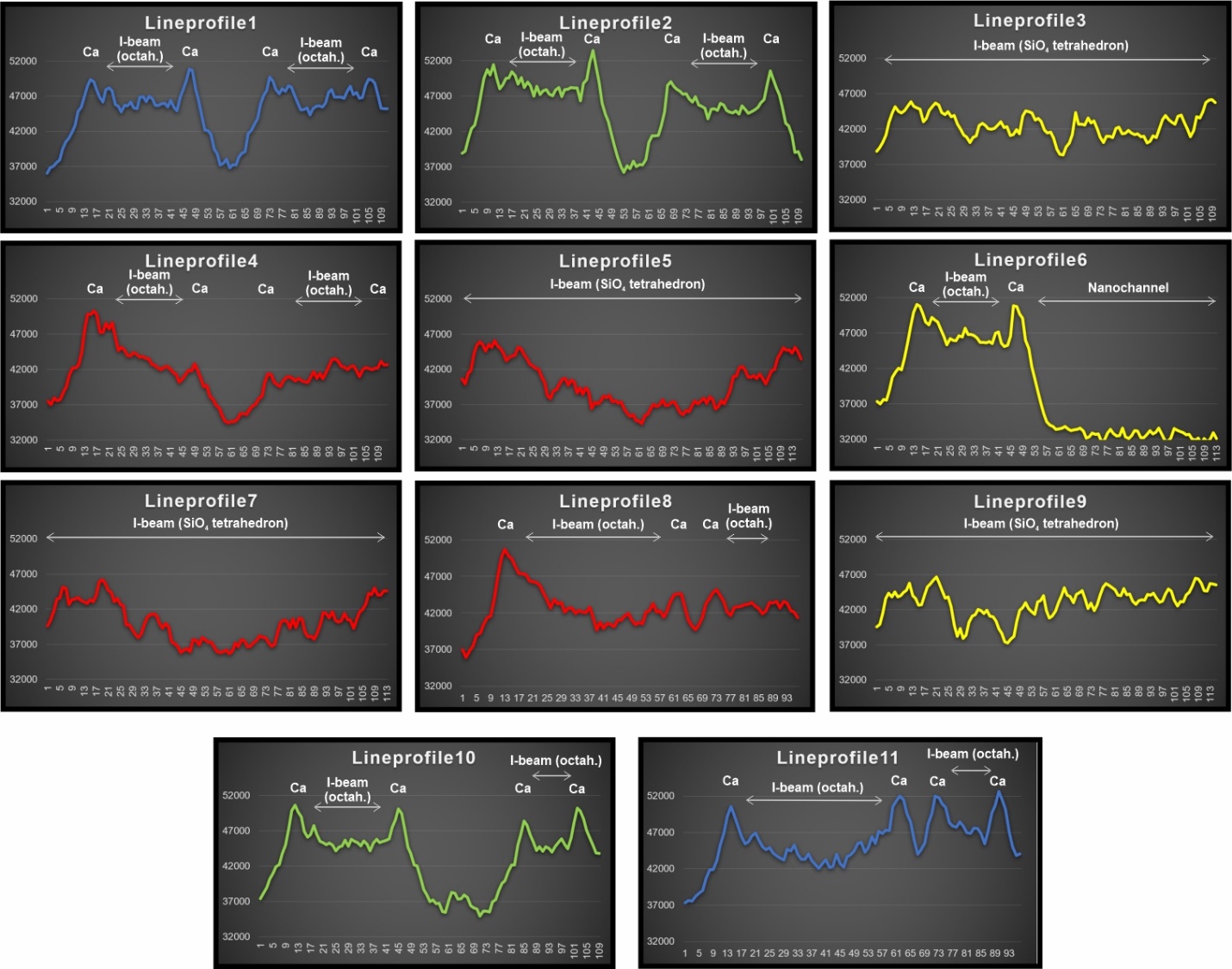


**Fig. S5.** Intensity profiles of **Supplementary Fig. S4.** All intensity profiles are parallel to the (100) even-odd interface. The amphibole (1-6), pyribole (7-11) and clinopyroxene (7-11) I-beams. Profile 6 penetrates the centre of the nanochannel. Calcium shows the highest intensity peaks in the profiles (e.g., Lineprofile2,10), whereas the lowest intensity is observed for the nanochannel (Lineprofile6) and for the A position within of the amphibole (e.g., Lineprofile10). The I beam octahedral positions form a plateau between the Ca atoms (e.g., Lineprofile10) and shows higher reflection than SiO_4_ tetrahedron (Lineprofile3,9). Octah. = octahedral.

**6. Mechanism of the formation of a negative crystal shaped fluid inclusion**

Process of modal metasomatism governs composition change of the lithospheric mantle resulting in new mineral phases (e.g., [1]). Supercritical fluids play a key role in element transport and during supercritical fluid infiltration the bulk content of the lithospheric mantle changes. Fluid migration can occur through diffusion controlled migration and fluid vein formation (e.g., [1]). Latter process occurs through rock crack formation as a result of fluid overpressure that dominantly results in mineral cracking (e.g., [1]). Mineral cracking is followed by mineral crack healing that due to non-perfect healing results in entrapped fluid ( **Fig. S6**). According to experimental geochemical studies, entrapped fluid will re-equilibrate with the host mineral by decreasing the fluid-host mineral surface to the smallest surface area that corresponds to the negative crystal shape ([1]). Re-equilibration speed increases with temperature and, thus, lithospheric mantle minerals re-equilibrate under lithospheric mantle conditions in several days. The time of re-equilibration of lithospheric mantle minerals under surface conditions is almost infinite and, therefore, makes it possible to distinguish fluid inclusions formed in the lithospheric mantle from those that formed during xenolith uplift. During fluid inclusion re-equilibration within the former fluid filled area as a decrepitation halo, that surrounds the negative crystal shapes fluid inclusion crystal defects and entrapped nano-fluid inclusions can form ([1]).

**
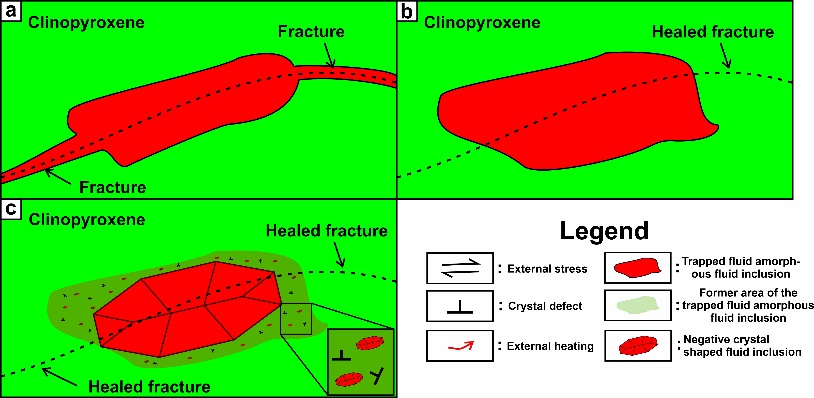
**

**Fig. S6.** Negative crystal formation showing different stages from (**a**) mineral cracking (**b**) though fracture healing and (**c**) negative crystal formation.

**References**

[1] O’Reilly, S. Y., & Griffin, W. L. Mantle metasomatism. Metasomatism and the chemical transformation of rock, 471-533 (2013).

[2] Bodnar, R. J., Reequilibration of fluid inclusions. In I. Samson, A. Anderson, & D. Marshall, eds. Fluid Inclusions: Analysis and Interpretation. Mineral. Assoc. Canada, Short Course **32**, 213-230 (2003).

[3] Bakker, R. J., & Jansen, J. B. H. A mechanism for preferential H_2_O leakage from fluid inclusions in quartz, based on TEM observations. Contributions to Mineralogy and Petrology, **116(1)**, 7-20 (1994).

**7. Calculating the composition of the pyribole and the pyribole to amphibole transformation**

For the calculation of the pyribole composition, we use the diopside and pargasite composition end-members as these are the most abundant molecules of our studied clinopyroxene and amphibole in xenolith PGR-X1-0345 ([1]). As the amphibole can be defined as a ‘PMP’ (Pyroxene-Mica-Pyroxene) sequence ([2]), the composition of the unknown ‘M’(Mica) can be calculated by extracting two times the diopside composition from the pargasite composition ([3]). Considering the above stated compositions, we get that ‘M’ composition corresponds to the preiswerkite (Na(Mg_2_Al)[Si_2_Al_2_O_10_](OH)_2_) mica composition. Based on the pyribole length and structure observed in the high-angle annular dark field images, we propose a PMMP sequence for the pyribole that is in agreement with the sequence of Veblen ([2]). Adding up 2 pyroxene (P) and 2 mica (M) compositions we acquire a composition of the Na_2_Ca_2_(Mg_6_Al_2_)[Si_8_Al_4_O_32_](OH)_4_ for the pyribole.

The difference between the pyribole and amphibole, in regard to their sequences, is one M. The length and position of the pyribole on the high-angle annular dark field images assume that two amphiboles form during pyribole to amphibole phase transformation. This suggest that 2 pyroxene (P) molecules should be added to the pyribole composition. This will result in the bulk composition of Na_2_Ca_4_(Mg_8_Al_2_)[Si_12_Al_4_O_44_](OH)_4_ that is equivalent to two amphibole molecules (NaCa_2_(Mg_4_Al)[Si_6_Al_2_O_22_](OH)_2_).

**References**

[1] Lange, T. P., et al. Nanoscale hydrous silicate melt inclusions at the clinopyroxene-amphibole interface in a mantle xenolith from the Perșani Mountains Volcanic Field. Lithos, **454**, 107210. <https://doi.org/10.1016/j.lithos.2023.107210> (2023a).

[2] Thompson, J. B. Biopyriboles and polysomatic series. American Mineralogist, **63(3-4)**, 239-249 (1978).

[3] Veblen, D. R. Non-classical pyriboles and polysomatic reactions in biopyriboles. Amphiboles and Other Hydrous Pyriboles—Mineralogy A, **9**, 189-236. <https://doi.org/10.1515/9781501508219-008> (1981).
